# Research Progress of Vertical Channel Thin Film Transistor Device

**DOI:** 10.3390/s23146623

**Published:** 2023-07-23

**Authors:** Benxiao Sun, Huixue Huang, Pan Wen, Meng Xu, Cong Peng, Longlong Chen, Xifeng Li, Jianhua Zhang

**Affiliations:** 1 School of Microelectronics, Shanghai University, Shanghai 201800, China; sunbenxiao@shu.edu.cn (B.S.);; 2 Key Laboratory of Advanced Display and System Applications, Ministry of Education, Shanghai University, Shanghai 200072, China

**Keywords:** ultra-high-resolution, flexible display, miniaturization, vertical channel, application in sensing, thin film transistor, photo lithography

## Abstract

Thin film transistors (TFTs) as the core devices for displays, are widely used in various fields including ultra-high-resolution displays, flexible displays, wearable electronic skins and memory devices, especially in terms of sensors. TFTs have now started to move towards miniaturization. Similarly to MOSFETs problem, traditional planar structure TFTs have difficulty in reducing the channel’s length sub-1μm under the existing photolithography technology. Vertical channel thin film transistors (V-TFTs) are proposed. It is an effective solution to overcome the miniaturization limit of traditional planar TFTs. So, we summarize the different aspects of VTFTs. Firstly, this paper introduces the structure types, key parameters, and the impact of different preparation methods in devices of V-TFTs. Secondly, an overview of the research progress of V-TFTs’ active layer materials in recent years, the characteristics of V-TFTs and their application in examples has proved the enormous application potential of V-TFT in sensing. Finally, in addition to the advantages of V-TFTs, the current technical challenge and their potential solutions are put forward, and the future development trend of this new structure of V-TFTs is proposed.

## 1. Introduction

The new technological revolution and continuous socio-economic development have led to rapid progress in new planar panel display technology. The demand for display and sensing technology is increasing.

Display and sensing technology has begun to develop towards ultra-high-resolution [1,2,3], low power consumption, large size, and flexibility [4,5,6,7,8,9,10]. In addition, the thin film transistor (TFT) plays a significant role as the core unit device. TFT is increasingly used in many electronic devices. The rapid updates and upgrades of these related electronic devices and products are inseparable from the progress of TFT technology in electrical performance, material, and process optimization [11,12,13,14].

As we all know, the function of sensors is to convert external signals into identifiable electrical signals. The indicators for evaluating the performance of sensors generally include sensing sensitivity, sensing resolution, triggering sensitivity, and so on. Based on our research experience in sensors, such as piezoresistive, or capacitive sensors, these basic sensing units may be limited in terms of inspection range or sensitivity due to their own material characteristics. Currently, a relatively simple method is to integrate thin film transistor (TFT) with sensors to form active sensors, improving the overall performance of sensor components. By integrating sensors with thin film transistor processes, the geometric dimensions of sensor devices can be reduced, and higher dimensional resolution sensor arrays can be manufactured. Therefore, the development of smaller TFT devices is of great significance to further reduce the geometry of TFT-sensor devices.

However, the problems encountered in the development of TFTs are very similar to those encountered in MOSFETs [15,16,17,18,19,20,21,22,23,24], to reduce the TFT footprint, achieve high-resolution, good sensing requirements, and improve integration density, it is necessary to reduce the TFT length of the channel. The reduction of channel length in traditional planar TFT is limited by the photolithography tools used in TFT manufacturing equipment, making it difficult to reduce the channel size of TFT to the sub-1 μm size.

A novel three-dimensional TFT structure, vertical channel thin film transistor (V-TFT) has been proposed. Compared to traditional planar TFT structures, this new type of TFT structure can effectively solve a series of problems caused by the reduction of TFT device size, under existing photolithography accuracy conditions, V-TFT can easily achieve sub-1μm channel lengths, achieve larger aspect ratios W/L, and higher packaging density, greatly reducing the occupied area of TFT devices. The application of this V-TFT to sensors will result in a significant increase in the vast majority of the sensing performance. Because the V-TFTs have a shorter channel used in sensors, due to the shortening of the channel, the carrier transport speed in the same conditions will be faster. Thus, shorter channel TFT preparation of the sensing sensitivity will result in faster sensing speed and better trigger sensitivity.

**Figure 1:**
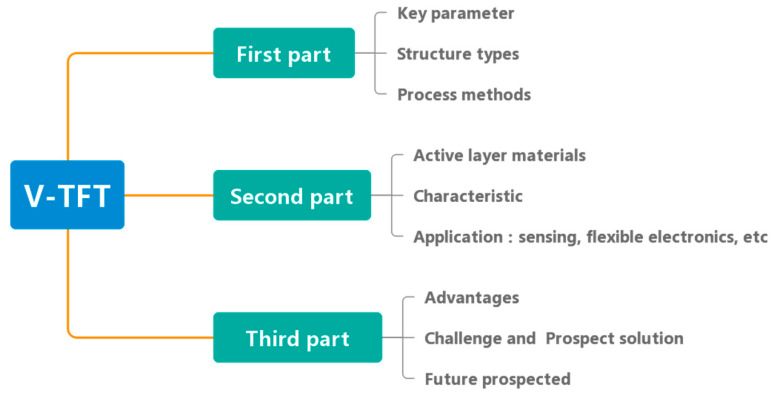
Logical framework for V-TFT introduction.

V-TFT has many advantages that planar devices cannot achieve, studying and improving the structure and material properties of V-TFT is of great significance with the development of next-generation ultra-high-resolution displays, flexible displays, and low-power sensor devices. The first part of this review was written to comprehensively elaborate on the research progress of V-TFT, including the structure types, key parameters, different preparation process methods and the spacer layer. The second part provides an overview of V-TFT active layer materials in recent years, and the characteristics of their applications using examples are considered. The last part provides an overview of the applications of TFT in sensors, and the advantages of V-TFT’s application in sensors is considered. This is followed by a brief summary of other applications of V-TFT, such as advanced displays, flexible electronics and memory. We summarize the problems and solutions faced by the overall development and further prospects for the future development direction of V-TFT. Figure 1 shows the overall Logical framework of the article.

## 2. V-TFT Device Key Parameters

In V-TFT devices, the key parameters for evaluating the electrical performance include the carrier mobility (μ), subthreshold swing (SS), and on-off current ratio (I_on_/I_off_). Therefore, in this section, we will provide a detailed introduction to these parameters. 

Firstly, we will introduce mobility. Regardless of V-TFT or planar TFT, the core component is the active semiconductor layer. However, the carrier mobility in TFT devices cannot be simply equated to a single-layer semiconductor active layer because the semiconductor active layer in TFT devices is affected by interface states. In V-TFT, the active semiconductor layer is mainly affected by the spacer layer, source electrode, drain electrode, and gate insulator layer. Therefore, using only electron or hole mobility cannot accurately describe the carrier movement in the device. In actual device research and preparation, mobility can be extracted from the saturation region and linear region of the transfer characteristics, corresponding to two different carrier mobilities. When extracting the mobility from the saturation region of the transfer characteristics, the mobility is composed of transfer curves at a larger V_ds_, and the slope of the transfer curve (g_m_) can be expressed as:

(1) $$g_{m}=\frac{\partial (\sqrt{I_{ds}})}{\partial (V_{gs})}|(V_{\mathrm{ds}}=\mathrm{constant})={\left(\frac{W}{L}\frac{C_{ox}\mu_{sat}}{2}\right)}^{\frac{1}{2}}$$

A slight variation of Equation (1) gives an expression for the saturation mobility:

(2) $$\mu_{sat}=\frac{2L{g_{m}}^{2}}{WC_{\mathrm{ox}}}$$

When the mobility is extracted in the linear region of the transfer characteristic, the mobility consists of the transfer curve at smaller V_ds_, and the slope of the transfer curve, g_m_, can be expressed as:

(3) $$g_{m}=\frac{\partial (I_{ds})}{\partial (V_{gs})}|(V_{\mathrm{ds}}=\mathrm{constant})=\frac{W}{L}C_{ox}\mu_{\mathrm{FE}}V_{\mathrm{ds}}$$

A slight variation of Equation (3) gives an expression for the mobility of the linear region:

(4) $$\mu_{\mathrm{FE}}=\frac{Lg_{m}}{WC_{\mathrm{ox}}}V_{\mathrm{ds}}$$

In the active semiconductor layer of a V-TFT, the mobility μ is subject to a variety of scattering regimes, including lattice vibrations, grain boundary effects, and other defect states such as doping impurities. In summary, the mobility of a V-TFT is the average rate of carrier movement per unit of electric field strength, which directly affects the operating frequency and operating current of the V-TFT device, and it is particularly important to adopt the correct method to extract and calculate the mobility of the device.

In addition, the subthreshold swing of the V-TFT is a particularly important device parameter. The subthreshold swing reflects the specific characteristics of the transition region from the off-state to the on-state in a V-TFT device. It is related to the density of defect states (subgap traps) in the band gap at the Fermi energy level [25], and the subthreshold swing can be expressed as:

(5) $$\mathrm{SS}=\ln\;10\frac{k_{B}T}{e}(1+\frac{eD_{sg}}{C_{G}})\;[\mathrm{meV}\;{\mathrm{decade}}^{-1}\;\mathrm{at}\;300\;K]$$

The specific definition of subthreshold is:

(6) $$\mathrm{SS}={\left\{\frac{d\log(I_{ds})}{dV_{gs}}\right\}}^{-1}$$

From Equation (6), SS is the increase in V_gs_ required for every ten-time increase in I_ds_ in the region of the transfer characteristic. The smaller the sub-threshold swing, the better the device performance of the V-TFT will be.

In the switching current ratio I_on_/I_off_, I_off_ is the tiny current between the source and drain when the V-TFT device is in the off-state. This current I_off_ is known as the off-state current and is also the minimum current I_ds_ in the characteristic transfer curve of the device. The parameter I_on_ is the maximum current value I_ds_ in the transfer characteristic curve when the V-TFT device is in the on state. The maximum I_ds_ is controlled by V_gs_ and V_ds_, but is also influenced by other factors, such as the size of the structure and the gate insulator. In summary, the switching current ratio I_on_/I_off_ is the ratio between the maximum I_ds_ and the minimum I_ds_ in the device’s transfer characteristic curve, and it reflects the switching capability of the device. The higher the switching current ratio, the better the relative performance of the device.

## 3. The Structure of V-TFT Devices

The V-TFT structure is different from traditional planar structure. Generally speaking, the source electrode and drain electrode of the V-TFT structure are not in the same plane, they separate the source electrode and drain electrode vertically using a thin layer of insulator material called a spacer, the channel is located between the source electrode and the drain electrode, which is attached to the vertical sidewall of the spacer layer, and the flow direction of conductive carriers is in the vertical direction. That is the channel carrier flow direction is perpendicular to the horizontal substrate direction, so it is called V-TFT.

At present, the V-TFT structure with the most reports will have a separate layer of material explicitly used as the spacer layer for the device. The mesa structure is the most representative, Ahn et al. reported a mesa structure [26], in this basic mesa structure, the size of the source electrode and the drain electrode is inconsistent, the second deposited electrode is deposited after the spacer layer material, aligning the electrode size with the spacer layer material size. Afterwards, the active layer, gate insulator (GI) layer, and gate electrode are deposited sequentially after the drain electrode.

Ryoo et al. prepared a structure similar to a “double gate” by sharing the source electrode and drain electrode [27], the structure can be equivalent to two V-TFTs in parallel, and the two end gate control the channel on each side to conduct the carrier. This type of “double gate” structure will save more space compared to the basic mesa structure when all other preparations are the same.

**Figure 2:**
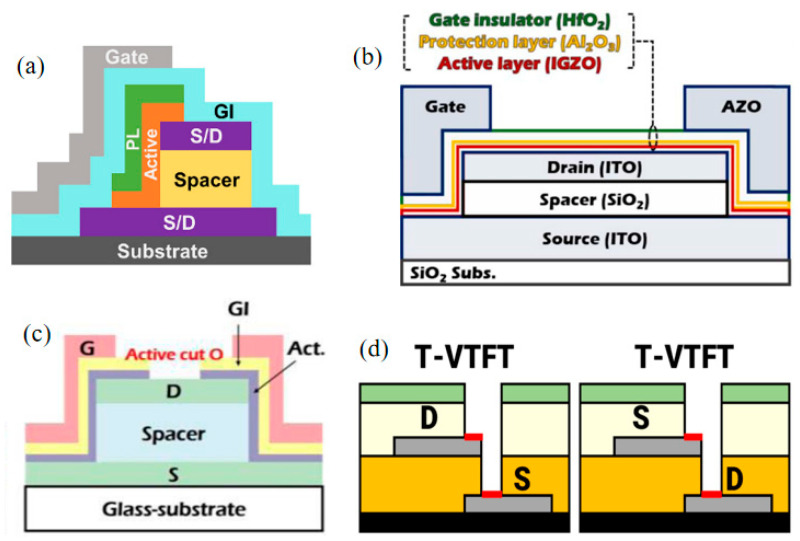
(**a**) Schematic diagram of mesa-shaped vertical structure [26]. (**b**) “Double gate” vertical structure sharing S and D electrodes [27]. (**c**) “Active-cut” vertical structure [28]. (**d**) Schematic Diagram of Trench Vertical Structure [29].

Kim et al. prepared another “double gate” structure [28], and proposed a vertical structure of “active cut” by “cutting off” the previous gate insulator layer and the active layer. This “active cut” structure is compared to a normal structure; it has higher mobility and a higher switching ratio, which can better avoid the floating body effect of devices and effectively reduce the off-state current. Figure 2 show the different V-TFT structure.

Based on these vertical structures, Ahn et al. proposed a trench-type vertical structure [29]. Unlike the mesa structure, in this trench-type vertical structure the source electrode and drain electrode have the same size. During the process of etching to form a vertical channel sidewall, the shape is similar to a trench type. Hence it is called a trench-type vertical structure, and the schematic diagram is shown in Figure 2d, this structure is compared to a mesa vertical structure. The test results indicate a higher mobility, it can effectively suppress the impact of Drain Induced Barrier Lowering (DIBL) on the device.

**Figure 3:**
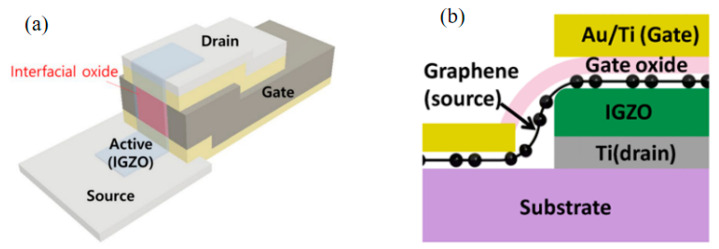
(**a**) The vertical structure of the gate electrode as a spacer layer [30]. (**b**) The active layer itself serves as the vertical structure of the spacer layer [32].

The V-TFT structure mentioned above utilizes a spacer layer to separate the source and drain electrodes, creating a vertical structure. Additionally, there have been reports of using gate electrodes to replace spacer layers for this purpose [30,31]. As shown in Figure 3a, in the structure proposed by Baek et al. [30], the interface between the gate electrode, the source electrode, and the drain electrode has a growing interlayer dielectric (ILD), to block the electrical interconnection between metals. This structure does not require the deposition of an additional spacer layer in addition to depositing three types of electrodes. The channel length of this V-TFT is determined by the thickness of the deposited gate electrode.

There is also a structure using In-Ga-Zn-O (IGZO) as the active layer, where the active layer separates the source electrode from the drain electrode in the vertical plane and the active layer itself acts as a spacer layer [32,33]. Figure 3b show the schematic diagram. This special vertical structure is based on the graphene-IGZO junction, utilizing the advantages of graphene’s high mechanical strength and high transport current make the conductive direction of the charge carriers in the active layer all perpendicular to the substrate direction, exhibiting high switching current ratio and high current density. The unique vertical transistor architecture can readily enable ultrashort channel devices with very high delivering current and exceptional mechanical flexibility. The channel length of this V-TFT is determined by the thickness of the deposited semiconductor active layer.

## 4. V-TFT Preparation Process and Spacer Material Selection

The preparation process of V-TFT is similar to that of traditional planar TFT. The vast majority of equipment is universal, physical vapor deposition (PVD), plasma enhanced chemical vapor deposition (PECVD), atomic layer deposition (ALD), solution spin-coated, the source electrode, the drain electrode, and the gate electrode of the deposition device are mainly used.

For devices, individual depositing of the source electrode and drain electrode by spacer materials separate. When preparing the source electrode of V-TFT, PVD is usually used to first deposit electrode film material which immune in dry etching. This purpose is to prevent the electrode material from being over etched during subsequent dry etching of other metal electrodes and spacer layers. A source electrode is formed by photolithography, development, etching, and patterning of electrodes.

**Figure 4:**
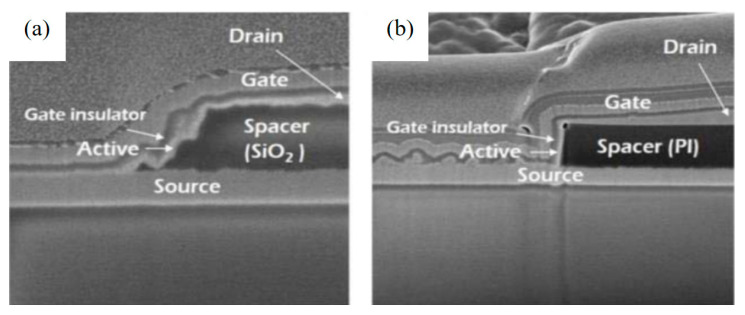
FIB-SEM images using (**a**) PECVD-grown SiO_2_ and (**b**) spin-coated PI spacers [28].

After depositing the source electrode, a spacer material layer is deposited using PECVD, or a solution spin-coated method is used to spin-coat a spacer material layer. Kim et al. found [28] that inorganic materials deposited by PECVD and organic materials spin-coated by solution also have a certain impact on the vertical angle of the formed vertical sidewalls, as shown in Figure 4a,b. The device uses solution spin-coated PI as the spacer layer. The closer the vertical angle of the device channel formed is to 90°, the better the device’s performance.

**Figure 5:**
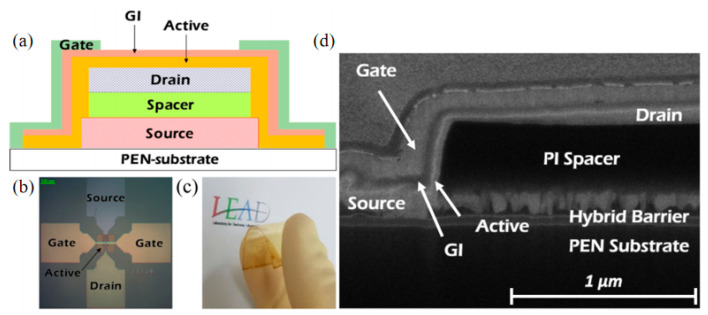
(**a**) Cross sectional view of flexible V-TFT. (**b**) Preparing the layout designed for V-TFT. (**c**) Schematic diagram after delaminated from the glass substrate. (**d**) Device image of FIB-SEM [34].

Afterwards, Hyeong Rae Kim from the same research group used plastic PEN material as a flexible substrate, and spin coated PI as spacer material using a solution spin coating method to prepare a flexible V-TFT that can bend and fold [34]. The V-TFT prepared using this method also has a vertical sidewall close to 90°, providing a very useful reference value for the miniaturization, bending, and scalability of flexible electron sensors. Figure 5a–c shows a cross-sectional view of this V-TFT device preparation, a layout, and an elastic bending schematic delaminated from the glass plate, respectively, and Figure 5d shows an FIB-SEM image of the prepared V-TFT.

In the selection of spacer materials, different factors need to be taken into account, such as insulation properties, process preparation compatibility, surface properties of the material and the cost feasibility of preparing the material. Here we present the selection criteria in a simple table, see Table 1. However, in the actual preparation and further development of the V-TFT, more factors need to be taken into account in order to develop a better performing V-TFT device. Here we are just illustrating a few of the more critical factors.

The thickness of the spacer during deposition or spin-coating is also a question worth studying. To achieve the advantages of V-TFT compared to traditional planar TFT, the spacer thickness is less than 1 μm, mostly between 100 nm and 500 nm [27,28,29,34]. One can usse PVD to deposit a layer of metal film on the spacer as a drain electrode film. The drain electrode is also formed through photolithography, development, and etching patterning.

At this time, patterning the spacer is the most important step in forming a vertical sidewall. The spacer was dry etched using reactive ion etching (RIE) in dry etching. By controlling the dry etching time, a vertical sidewall was ultimately formed. Afterward, the deposition of the V-TFT stack structure was initiated, consisting of three parts: the active layer, the gate insulator layer, and the gate electrode. Using PVD or ALD to deposit active layer materials above the active layer, the active layer is formed through photolithography, development, and etching patterning. Afterward, the same method is used to deposit the gate insulator layer and gate electrode. In relevant reports it was found that [35,36,37] when using ALD to deposit all layers, the V-TFT device formed a more obvious vertical sidewall profile when forming a vertical sidewall. This is due to the high conformability of ALD.

**Figure 6:**
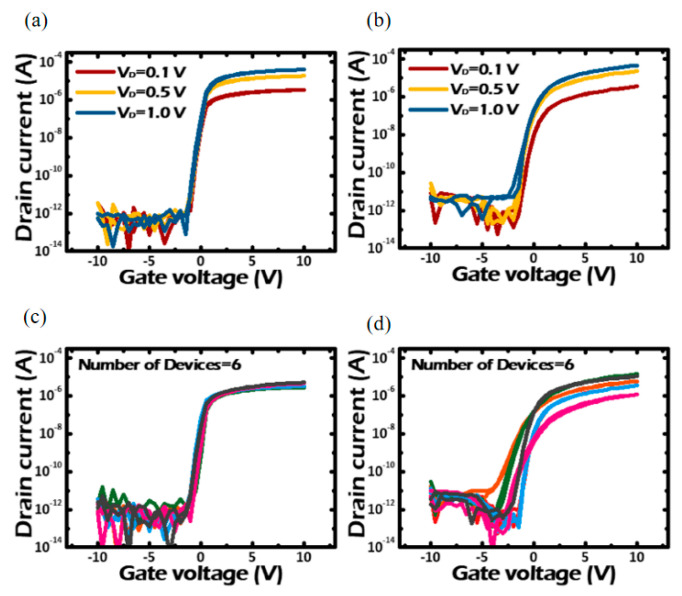
V−TFT transfer characteristic (I_ds_−V_gs_) prepared by different deposition methods (**a**) ALD deposition (**b**) sputtering deposition (**c**) transfer characteristic changes of 6 devices deposited by ALD (**d**) transfer characteristic changes of 6 devices deposited by sputtering [36].

Figure 6 shows a graph of the comparison between using an ALD deposited stack structure and using a sputter deposited stack structure. It is clear from Figure 6 that the device performance of the ALD deposited stack structure is significantly better than the sputter deposited stack structure.

After the formation of the vertical sidewall, in addition to the main conductive current path formed at the interface, the area between the gate insulator layer and the active layer also forms the current path. At the interface between the active layer of V-TFT and the spacer layer that separates the source electrode and drain electrode, a conductive current path parallel to the main current is also formed, which is called the back channel current. This interface is called a back channel interface. The leak electrode passes through the spacer layer in the resulting electric field line, causing it to collect charges at the interface between the active layer and the spacer layer. Under off-state conditions, this kind of back channel current is the main factor of the drain leakage current.

The gate capacitance per unit area increases with the decrease of the gate insulator layer. At this time, the equivalent capacitance at any point at the interface between the gate electrode and the back channel increases. That is, the gate capacitance per unit area increases. If the parasitic resistance with smaller values is ignored, the voltage at any point at the interface between the active layer and spacer can be expressed as [38]:

(7) $$V_{\mathrm{bg}}=V_{\mathrm{dg}}\left(\frac{C_{\mathrm{bd}}}{C_{\mathrm{bd}}+C_{\mathrm{eq}}}\right)$$

V_bg_ represents the voltage value between the back channel interface and the gate electrode, V_dg_ represents the voltage value between the drain electrode and the gate electrode, C_bd_ represents the capacitance value between the back channel interface and the drain electrode and the C_eq_ represents the equivalent capacitance value per unit area. From Equation (7), it can be seen that as the equivalent capacitance of the gate increases, the correlation between the back channel voltage and the drain electrode bias will decrease. From this, it can be seen that a decrease in the thickness of the gate insulator layer will reduce the drain electrode current, as a decrease in the thickness of the gate insulation layer will enhance the gate’s ability to control the back channel.

The gate serves as the spacer, or the active layer itself serves as the vertical structure of the spacer, and its preparation process is similar to the above method, except for different deposition sequences and types of materials used. There are still many areas for improvement in the V-TFT process method mentioned above. As a new type of TFT device structure, the unique nature of its structure leads to some common problems in the process of V-TFT, such as the back channel effect [39,40]. Due to the back channel effect of V-TFT, its leakage current is higher; in 2021, the Sung et al. research group conducted a detailed analysis of the impact of the back channel effect [41], and a detailed study was conducted on the elements between the channel layer and spacer layer using time-of-flight secondary ion mass spectrometry (ToF-SIMS).

**Figure 7:**
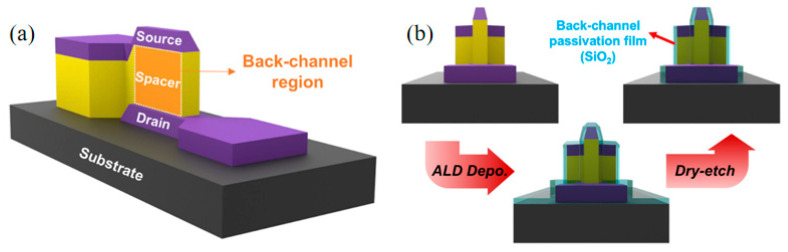
(**a**) Schematic diagram of the back channel region in V-TFT. (**b**) Schematic diagram of protection treatment for the back channel region [26].

During the etching of the spacer layer, a large number of impurities remain in the back channel area; there are a large number of interface and defect states, and this rough vertical sidewall can cause an increase in the off-state current of the device. Therefore, further optimization of the process is one of the key factors in the development of V-TFT. Lee et al. proposed a solution to the problem of the back channel effect [26]; after patterning the back channel, a layer of SiO_2_ was deposited using ALD as the protection layer (PL) and patterned. Compared with devices without a protective layer, V-TFT with a protective layer significantly reduces its subthreshold swing (SS). Figure 7 is a schematic diagram of this method.

In addition, by setting a reasonable spacer thickness and adjusting its process parameters, the prepared V-TFT can achieve a lower leakage current of the drain electrode. TFT and MOSFET belong to the metal-insulator semiconductor field effect transistor (MISFET) three-terminal device structure. According to the Gradual channel approximation (GCA) equation [42]:

(8) $$I_{d}=\frac{W}{L}\cdot \frac{\mu C_{\mathrm{ox}}}{2}\left[2\left(V_{\mathrm{gs}}-V_{\mathrm{th}}\right)V_{\mathrm{ds}}-{V_{\mathrm{ds}}}^{2}\right]$$

I_d_ is the drain driving current, W/L is the width to length ratio of the channel, μ is the device mobility, C_ox_ is the per unit gate oxide layer capacitance. V_gs_, V_th_ and V_ds_ are the voltage between the gate electrode and the source electrode, and the voltage between the source electrode and the drain electrode. According to Equation (8), the drain driving current is inversely proportional to the length of the channel. Under certain conditions, the shorter the channel length of the device, the greater the drain driving current. In V-TFT devices, by adjusting the channel length and ensuring appropriate process conditions, a larger drain driving current will be obtained. Therefore, reasonable regulation and exploration of the preparation process of V-TFT is a topic worthy of further research.

## 5. The Development of V-TFT Active Layer Materials

Similar to planar TFT devices, the development of the active layer of V-TFT has followed the changes of amorphous silicon (a-Si), low temperature polycrystalline silicon (LTPS) and amorphous oxide semiconductor (AOS), but there is still much to explore in the change of the active layer of V-TFT.

### 5.1. a-Si as V-TFT Active Layer

As early as the end of the last century, inspired by the development of MOSFETs toward three-dimensional structures, Uchida et al. proposed the structure of V-TFT in 1984 [43]. Then in 1997, based on the excimer laser method, his team used a-Si as the active layer to prepare the V-TFT structure [31]. Due to the technical conditions at this time, the performance of the V-TFT device prepared could have been better. In 2005, Chan et al. prepared a 100 nm channel length V-TFT device using hydrogenated a-Si, with a unit area of one-third of the traditional planar TFT area [44].

### 5.2. Low Temperature Polycrystalline Silicon as V-TFT Active Layer

In devices with low-temperature polycrystalline silicon as the active layer, in 2008, Toure et al. prepared a quasi V-TFT structure by increasing the channel width at relatively low temperatures [45]. However, due to the channel width, the device size was too large. Later in 2013, their team optimized this quasi-V-TFT structure, which improved device performance. However, there were still issues with excessive overlapping areas of source and drain electrodes, resulting in high off-state current [46].

### 5.3. AOS and Other Materials as V-TFT Active Layers

In devices with amorphous oxide semiconductors (AOS) as active layers, there are various types of AOS. However, due to the relatively low cost and large mobility of ZnO and IGZO compared to other AOS, the selection of active layers for V-TFT is mostly focused on the research of ZnO and IGZO [47,48]. Moreover, due to the special structure of V-TFT, AOS is more advantageous as the active layer of V-TFT. That is, when forming a vertical sidewall structure, in addition to traditional sputtering such as PVD, AOS and gate insulator layers used for AOS (for example, Al_2_O_3_ or SiO_2_) can also be deposited using ALD with better conformal properties [49,50].

In the devices prepared with ZnO as the active layer in 2012, Nelson et al. used ZnO to prepare V-TFT devices [51]. Under the same preparation conditions, the mobility is ten times that of amorphous silicon. However, due to the fact that the gate serves as the spacer layer, the overlap capacitance between the gate electrode and the top source electrode is large. In the following year, their team applied the prepared V-TFT device to the ring oscillator, and the ring oscillator performed well [52]. This indicates that V-TFT also has great potential in circuit applications. In 2015, Sun et al. used spatial atomic layer deposition (SALD) to deposit ZnO as channel material [39], V-TFT devices with the gate as the spacer were prepared. By comparing the simulation and experimental results, the behavior of defects was simulated, and it was found that the defects were caused by the large defect state in the back channel. In 2016, Yeom et al. used the sputtering method to deposit IGZO and plasma enhanced atomic layer deposition (PEALD) to deposit InO_x_ as two different active layers of V-TFT. The V-TFT tested has a high turn on state current [53].

**Figure 8:**
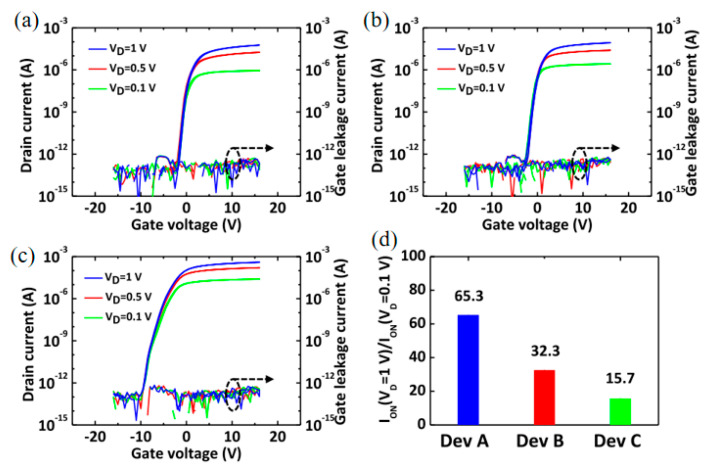
(**a**) Device type A transfer curves at different voltages. (**b**) Device type B transfer curve at different voltages. (**c**) Device type C transfer curve at different voltages. (**d**) Ratio of I_on_ at V_ds_ = 1 V to I_on_ at V_ds_ = 0.1 V for three devices [54].

From 2017 to 2023, Sung et al. conducted multiple research works on V-TFT prepared with IGZO as the active layer. This includes optimizing the structural design of V-TFT in the layout [54], selecting appropriate gate dielectric layer materials [27], optimizing the active layer, and so on. In the optimized active layer, the IGZO active layer suitable for V-TFT can be obtained by adjusting the different components of four elements in IGZO in ALD. Sung et al. deposited two different types of IGZO films in the vertical sidewall by adjusting the super cycle ratio of ALD to change the In, Ga, and Zn elements in IGZO, forming a double active layer [55]. Figure 8a–c shows the transfer curves of three types of V-TFT, ABC, by adjusting the composition of IGZO elements. The super cycle ratios of the three types of ABC are TEIn:In-Ga: DEZn = 0:2:2, TEIn:In-Ga: DEZn = 1:2:2, and TEIn:In-Ga: DEZn = 2:2:2. The atomic percentages of In, Ga, Zn, and O in ABC are 4:20:24:52, 10:14:26:50, and 15:11:22:52, respectively. By forming a two-dimensional electron gas junction on the back channel of V-TFT, the threshold voltage drift of the double active layer is greatly reduced compared with that of the single active layer.

**Figure 9:**
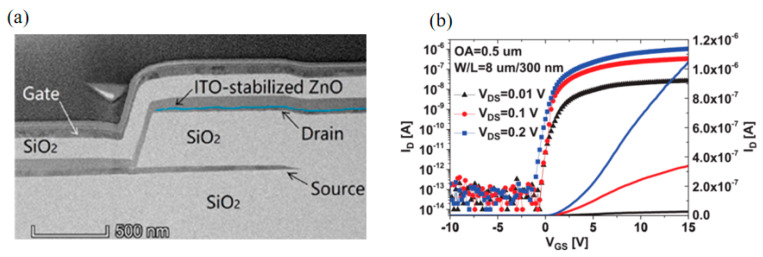
(**a**) TEM Image of V−TFT active layer with composite crystal ITO−ZnO. (**b**) Transfer characteristics of V−TFT active layer devices with composite crystal ITO−ZnO at different voltages [56].

In addition to using IGZO as the active layer, Yin et al. used co sputtering deposition of composite crystal ITO-ZnO as the active layer of V-TFT [56]. Their test found that the V-TFT structure with a designed overlap area (OA) of 0.5μm between the source electrode and drain electrode has high on/off current ratio performance, up to 10^7^. Figure 9a,b shows the TEM images of this V-TFT and the transfer characteristics of the device under different V_ds_ voltages.

In order to comprehensively show the development state of V-TFT in recent years, some representative V-TFT devices have been summarized. Table 2 summarizes the key parameters of V-TFT in recent years, including mobility μ, subthreshold swing, threshold voltage $V_{\mathrm{th}}$, current ratio $I_{\mathrm{on}}{/I}_{\mathrm{off}}$, materials used for depositing active layers, and their deposition methods. From Table 1, it can be seen that the progress and development of V-TFT are not linear, but rather a nonlinear development.

## 6. The Advantages and Practical Applications of V-TFT

V-TFT has a number of unique advantages over conventional planar TFT structures, we have summarized the advantages and details of V-TFT in Table 3, and the V-TFT has the following characteristic advantages:

(1) V-TFT structures are independent of the requirements of high-precision photolithography, precise control of the channel length by adjusting the thickness of the deposited film (spacer layer) allows easy access to sub-1 μm channel lengths. Independent of high-precision photolithography requirements, it will reduce significant costs.
(2) The shortening of the V-TFT channel length, which enables the requirement for a high-resolution TFT display and an increase in the corresponding pixel density. Higher package densities are obtained, and their integration is increased.
(3) The reduction of the V-TFT channel length enables the TFT width-to-length ratio W/L to increase, achieves lower power consumption, allows control of high currents at low voltages, and greater on-state currents under the same conditions.
(4) The channel structure of the V-TFT is on a vertical sidewall, and when the device itself is bent and stretched by the flexible substrate, the device will not be much affected by the bending of the substrate because the channel direction is perpendicular to the substrate. This will enable us to meet the needs of today’s society in terms of ultra-high-resolution displays and flexible displays.

**Table 3:** Summary of the advantages of V-TFT.

Device Type	Channel Aspects	Physics Aspects	Structure Aspects
Planar TFT	Lithography equipment limitations make it difficult to achieve sub-micron channel length	Difficulty in obtaining high current at low voltage, challenge in improving packaging density	The structure itself is greatly affected by bending
Vertical TFT	Independent from high precision photolithography requirements and easy access to sub-micron channel length	Smaller size, high-resolution, low power consumption, higher integration	Channel direction is perpendicular to the substrate, not affected by the bending of the substrate

**Figure 10:**
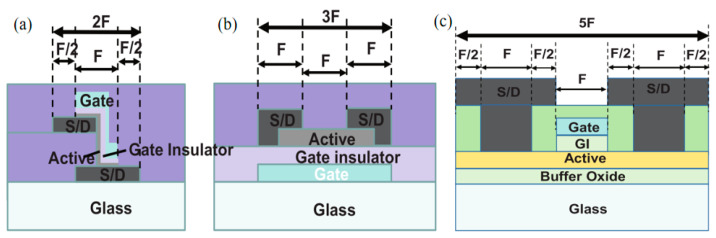
Cross section comparison of three different TFT structures: (**a**) V-TFT structure cross section diagram. (**b**) Back channel etching TFT structure cross section diagram. (**c**) Self-aligned TFT structure cross section diagram [53].

The greatest advantage is that V-TFT devices take up less area under the same conditions [53], this makes it valuable for a wide range of applications in ultra-high resolution displays and the miniaturization of devices. As shown in Figure 10, a cross section comparison of the V-TFT and conventional planar back channel etching (BCE) structured TFT, self-aligned structured TFT with their smallest individual device dimensions. A comparison of the three structure diagrams easily shows that the V-TFT has the smallest area. Most AMOLED use 2T1C circuits as ultra-high resolution display panels, where conventional planar TFTs are large in size, and short-channel TFTs should be selected. On the other hand, the short channel size of V-TFT is a unique advantage that will allow the application of this display panel when it is mature.

**Figure 11:**
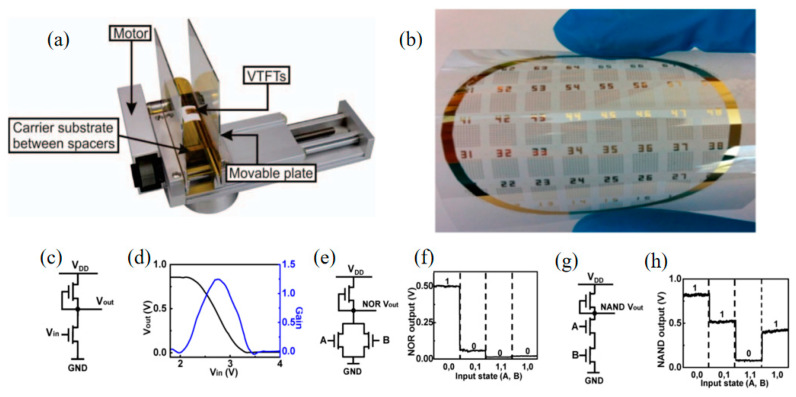
V-TFT applied to: (**a**) schematic diagram of testing under bending conditions [58]. (**b**) PET flexible substrate. (**c**) prepared inverter. (**d**) diagram of the relationship between the input and output of the prepared inverter and its gain. (**e**) prepared NOR logic gate. (**f**) four typical input-output state diagrams of prepared NOR logic gate. (**g**) schematic diagram of prepared NAND logic gate. (**h**) four typical input and output state diagrams of the prepared NAND logic gate [32].

Flexible electronics are also being used more and more in the display field [59]. Due to its unique structural characteristics, the V-TFT has its conducting channel carriers oriented perpendicular to the substrate. Its structure is hardly affected by substrate bending during the bending process. Yuan et al. have applied V-TFT to the flexible field and prepared this structure with logic circuits on substrates [32]. Simple circuits were prepared using the V-TFT, including inverters, NOR logic gate, and NAND logic gate etc. Figure 11 shows the schematic diagram of the circuit prepared and its input and output states’ diagrams.

**Figure 12:**
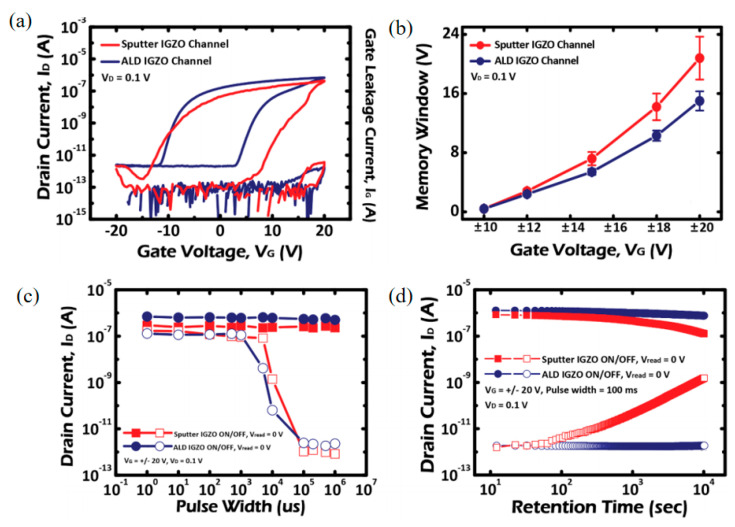
(**a**) I_ds_−V_gs_ characteristics and the (**b**) variations in MW width for the fabricated V−TFT charge−trap memory using sputtered and ALD−grown IGZO active channel layers. (**c**) Variations in the on- and off-programmed I_ds_’s of the fabricated V−TFT charge−trap memory (**d**) Variations in the on- and off-programmed I_ds_’s with a lapse of memory retention time for 10^4^ s at RT [37].

In addition, like MOSFET, three-dimensional structures are used in new storage technologies [61,62,63,64], V-TFT can also be used in the field of memory devices. In 2020, Kim et al. successfully applied the prepared V-TFT device structure to 3D NAND memory with a storage window of up to 15 V and a retention time of up to 10 years [37]. Figure 12a and Figure 12b respectively show the fabricated devices, including variations in MW width for the fabricated V-CTM TFT using sputtered and ALD-grow IGZO active channel layers. Figure 12c,d shows the relationship between the current of the V-TFT prepared memory and the variation of Retention time and Pulse width.

Otherwise, YANGTZE River Storage company used unique and innovative X-tacking storage architecture with 3D NAND [65], this structure also has a vertical channel conductivity direction, and vertical structures are used in memory devices, which is a major direction for future development. Combining V-TFT with 3D NAND storage technology, will lead to more meaningful development of device properties.

## 7. Huge Potential for V-TFT Applications in Sensing and Other Areas

**Figure 13:**
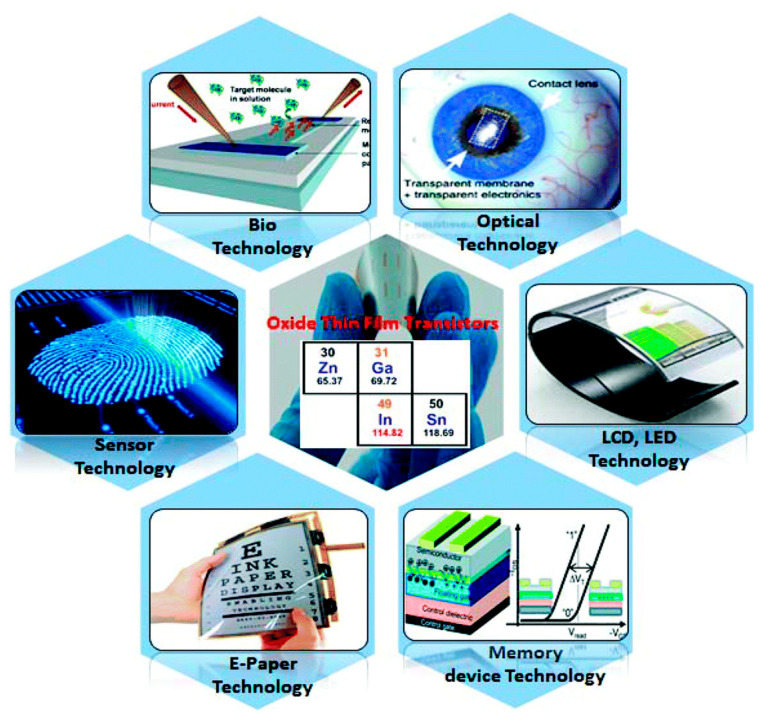
Application of TFT in flexible electronics sensing application [66].

Due to the fact that V-TFT is a new type of structural device, there are still few reports on V-TFT. However, V-TFT is one of the types of TFT that differs from conventional planar TFT structures only in that the conductive direction has been changed from a two-dimensional plane to a three-dimensional conductive direction. There are numerous reports of TFT applications in flexible electronics, as described in this paper’s introduction. Figure 13 illustrates a diagram of a TFT in a flexible electronics sensing application [66].

As can be seen in Figure 13, TFT has numerous applications in flexible electronics, including sensing technology, electronic paper, memory, LCD and LED, optoelectronics, and biotechnology. In particular, in sensing technology, TFT can be used in numerous areas of sensors [67,68,69,70,71,72,73,74,75,76,77,78], such as pressure sensors, biosensors, chemical sensors, etc. Next, we will introduce some specific examples of TFT applications in the field of sensors.

**Figure 14:**
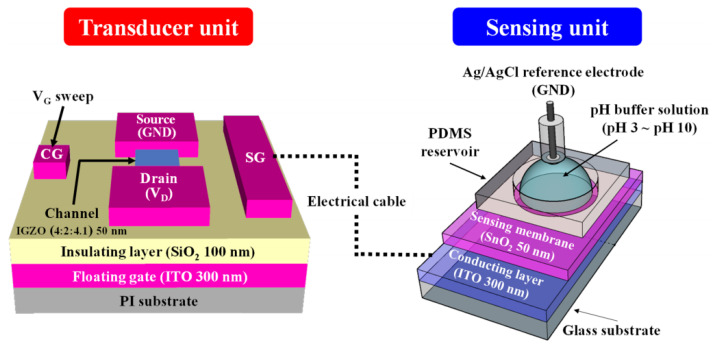
Schematic illustration of an a-IGZO coplanar dual-gate TFT transducer and SnO_2_ EG sensing units. The dotted line represents the electrical connection between the two units [79].

Tae-Hwan Hyun et al. proposed a completely transparent and flexible high-performance pH sensor based on an a-IGZO TFT transducer, which has a coplanar double grid structure on a polyimide substrate [79]. It was also verified experimentally that after 500 bending cycles, this TFT-based chemical sensing still exhibited good stable, and reliable high-sensitivity. Figure 14 shows the schematic diagram of this TFT-based chemical sensing. This offers a significant range of applications for TFT in PH detection sensors.

**Figure 15:**
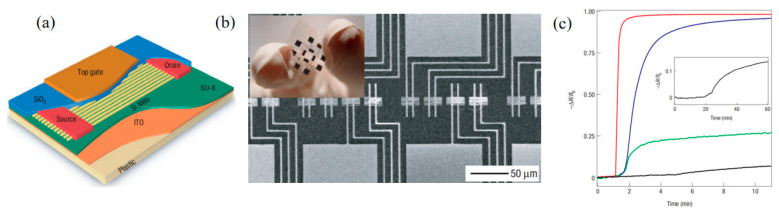
(**a**) Schematic illustration TFTs on plastic, with the electrodes and various layers labelled. (**b**) SEM image of an array of nanowire sensors based on TFT. Each device (horizontal strip) is contacted by two Ti electrodes (oriented vertically). Inset: Digital photograph of the flexible sensor chip. (**c**) Electrical response of a nanowire sensor to 20 p.p.m. (red curve), 2 p.p.m. (blue curve), 200 p.p.b. (green curve), and 20 p.p.b. (black curve) NO_2_ diluted in N_2_. The gas is introduced to the sensing chamber after 1 min of flowing N_2_. Inset: An extended response of the sensor to 20 p.p.b. NO_2_; the gas is introduced after 20 min of flowing N_2_ [80].

In addition to TFT-based chemical sensing for PH sensors, there are also TFT-based chemical sensing for toxic gas detection, such as NO_2_ toxic gas. McAlpine, M. C et al. use the TFT with excellent chemical sensors, and exhibit parts-per-billion detection NO_2_ toxic gas. These sensors also can distinguish acetone and hexane vapors via distributed responses. The excellent sensing performance coupled with bendable plastic could open up opportunities in portable, wearable, or even implantable sensors. Figure 15 shows the TFT schematic for the preparation of this chemical sensor, the flexible sensor chip, and SEM image of an array of nanowire sensors, which detect the chemical gas electrical curves, respectively. This shows that TFTs have great potential for detecting toxic chemical gases.

**Figure 16:**
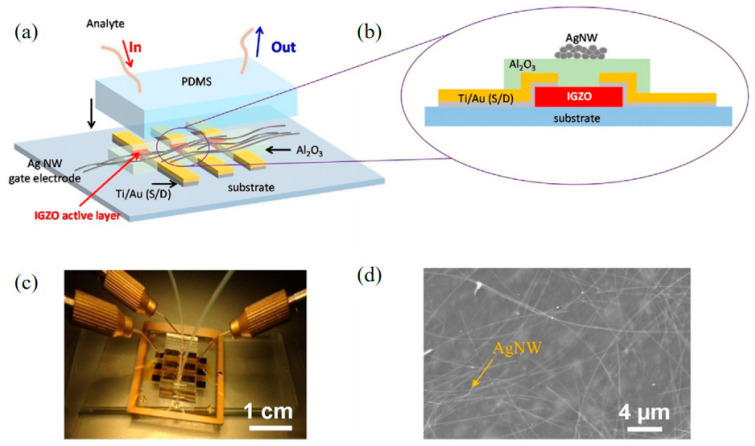
(**a**) Schematic of the TFT integrated in the PDMS chemical sensor. (**b**) The schematic view of IGZO TFT based sensor. (**c**) Photograph of the PDMS chemical sensor flow system. (**d**) SEM image of Ag NW mesh top gate electrode on IGZO TFT based sensor [81].

Based on TFT integration, chemical sensing can also detect other chemicals. Yoo, T.-H et al. use the Ag NW as the top gate electrode to prepare an electrode that could detect biologically relevant species such as H_2_O_2_, b-d-glucose, d-glucono-1,5-Lactione, and lactic acid in aqueous media [81]. This provides several ideas for further research using TFT integrated chemical sensors. Figure 16 shows the relevant schematic diagram of this chemical sensing.

**Figure 17:**
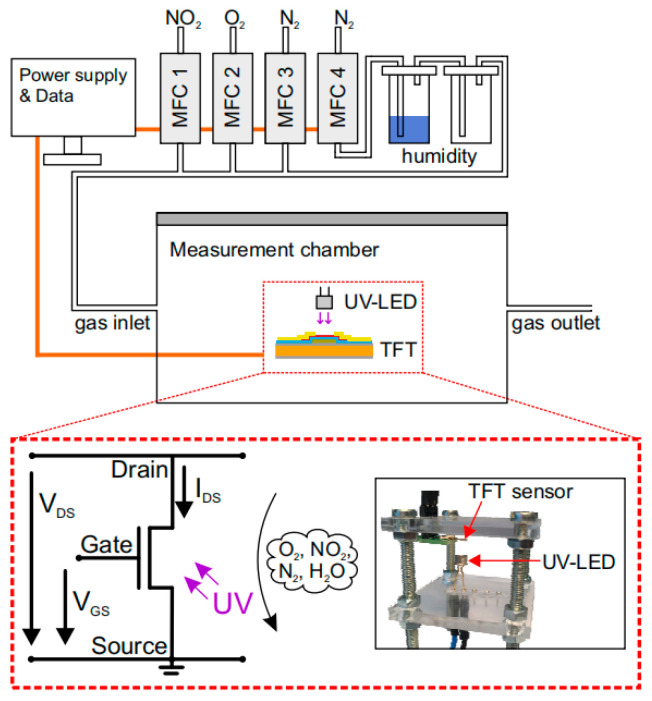
Schematic diagram of the working principle of a chemical sensor for the detection of different gases prepared by TFT integration. The desired gas mixture is prepared by four mass flow controllers (MFC 1–4), each connected to a different gas species [82].

Chemical sensing prepared using TFT integration can also be integrated into a large-scale sensing system. Stefan Knobelspies et al. fabricated gas chemical sensing on a free-standing flexible polyimide foil [82]. This chemical sensing can detect various trace gases. Figure 17 shows a diagram of how this TFT sensing system works.

**Figure 18:**
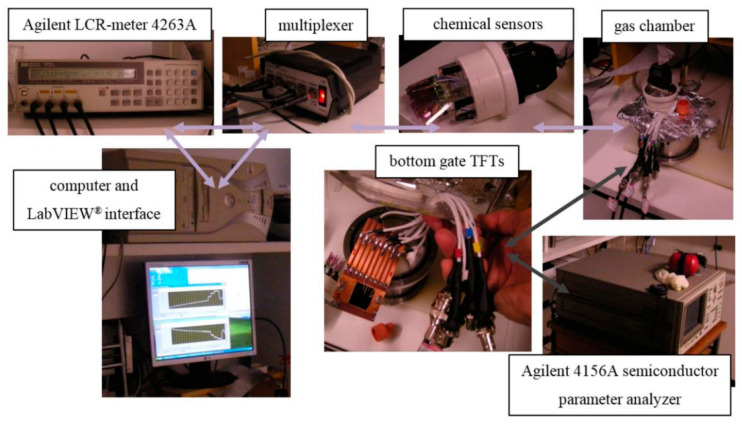
Gas detection system for chemical sensors and P3HT TFTs [83].

In addition, Marco R. Cavallari also reported a chemical sensor system based on TFT integration, which is based on P3HT TFT and can also accurately detect some gases. Figure 18 is a schematic diagram of this TFT integrated chemical sensor system.

**Figure 19:**
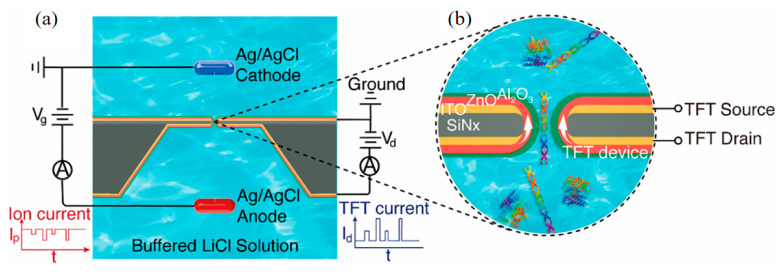
(**a**) Schematic of a V-TFT nanopore device and translocation experiment setup that concurrently measure both ionic and V-TFT signals. (**b**) Close-up schematic of the V-TFT nanopore device [84].

In terms of the specific application of V-TFT to sensors, Zhu, X et al. prepared V-TFT integrated into nanopores, and self-aligned nanoscale V-TFT integrated sensors can detect single bio-molecules for charge sensing [84]. In addition, using this V-TFT integrated nanopore sensor device, it is possible to detect both ion conductance blockade signals and TFT electrical current modulation signals. In this report, V-TFT integrated sensing to achieve charge-based single-biomolecular technology for basic research as well as for biosensing applications. Figure 19 shows a schematic diagram of how this V-TFT integrated sensor works.

In the previous section, we described the practical applications of TFTs for chemical and other types of sensing respectively. Each of these examples effectively demonstrates the wide range of applications for which TFT can be used to prepare sensors. 

In addition, the V-TFT has more potential for future applications in sensors. The unique vertical structure of the V-TFT, and its application in sensors will have the following advantages:

(1) V-TFT-based sensors are expected to have high sensitivity and high responsiveness. Because the V-TFT channel length is shorter compared to conventional TFT channels, the carrier transfer rate becomes faster under the same conditions, a characteristic that makes V-TFT-based sensors much more sensitive and responsive than planar TFT sensors under the same conditions.
(2) Since one of the advantages of V-TFT over conventional TFT is the small footprint, V-TFT-based sensors can offer unique advantages in higher resolution applications. In chemical sensors, this means being able to sense and measure changes in the concentration of target molecules in greater detail. The high resolution and precision provides more accurate analytical results and increases the sensitivity and reliability of the chemical sensor.
(3) Now, it has been reported that the V-TFT has a low leakage current [56]. In sensor applications, this also means that small current changes or signals can be measured more accurately, improving the sensitivity and accuracy of the sensor.

As an important structure of TFT, V-TFT has advantages that are not comparable to conventional planar structures. The unique characteristics of V-TFT can meet the needs of miniaturization development of chemical sensors mentioned above, achieving higher detection sensitivity in turn. In addition, with future developments, the preparation of inorganic and organic V-TFT will be integrated into a variety of sensors with a smaller size, smaller area, and lower power consumption than sensors integrated with conventional TFT.

In other potential applications, the biggest feature of V-TFT itself is its ability to miniaturize devices, which makes V-TFT applicable to various small devices in the future. Especially for AMOLED screens in ultra-high-resolution displays. The conductive structure itself is not affected by bending effects, and V-TFT can also be applied in flexible and wearable aspects.

## 8. Challenges and Prospects

There are many applications for TFT integration, but they are all trending towards miniaturization. These trends all require the TFT to be continuously decreased in size. As the size of TFT continues to decrease, the vertical channel thin film transistor is an effective solution to the problem of miniaturizing the size of conventional planar lateral channel TFT. 

Although V-TFT has many unique advantages compared to conventional planar devices, and some progress has been made in recent years, there are relatively few reports in V-TFT paper. In addition, its charge transport properties such as mobility and transfer could be lower than planar TFT, and I_off_ could be higher than planar TFT, these disadvantages are attributed to a number of urgent problems that need to be addressed:

(1) During the etching process, the vertical sidewalls have a rough surface with the unavoidable introduction of impurities, which in turn causes higher off-state currents.
(2) The reduced channel size of V-TFT devices will bring about the problem of the short-channel effect. To effectively suppress the short-channel effect, the thickness of the gate insulator layer needs to be reduced. The reduced in gate insulator thickness will inevitably lead to an increase in gate leakage current.

In addition, the process and choice of active layer material have a more significant impact on the performance of the V-TFT. For example, using a double active layer structure and including a protective layer in the vertical sidewalls can improve the V-TFT device’s performance. Searching for the proper process and active layer material is an essential prerequisite for preparing V-TFT devices with excellent performance.

The aforementioned issues result in overall inferior charge transport properties of V-TFT compared to traditional TFT. We have summarized a Table 4 comparing the charge transport properties of V-TFT and traditional TFT, clearly illustrating the differences. The below compares shows the planar TFT and vertical TFT of all the devices charge transport properties which use the IGZO as the active layer.

Although the current charge transport properties of V-TFT are not as good as traditional planar TFT, the development potential of V-TFT is enormous, and further research on V-TFT is needed to improve charge transport properties.

How to form good vertical sidewalls and selecting suitable gate dielectric and active layer materials is also a topic worthy of further research. The V-TFT still requires a long-term exploration process, performance optimization, and reliability. Overall, the V-TFT has many advantages, but at the same time, it also faces many challenging tasks. In terms of future development directions, the V-TFT can still learn from the development of MOSFETs, and the development of the vertical field effect transistor (VFET) is also being actively researched [92,93,94,95,96,97,98,99]. The emergence of FinFETs and CAA-FETs, which provide reference ideas for the development of V-TFT, such as the development of C-Axis Aligned Crystal TFT (CAAC-TFT) [100,101], which have a structure similar to that of CAA-FETs. With the development of various devices towards miniaturization, the V-TFT, due to its unique nature, will be of great advantage in the process of applying multiple miniaturization applications.

## 9. Conclusions

With the increasing demand for TFT technology in social applications, TFT technology is constantly being updated. Inspired by the vertical field effect transistor, the vertical thin film transistor (V-TFT) was developed to meet the requirements for low power consumption, large size, and low voltage control of high currents. V-TFT offers unique advantages compared with conventional planar TFT, but there are also many aspects for improvement. For example, how to form atomic-level conformality in the vertical sidewalls and form vertical sidewalls close to 90°, how to form good contact between the active layer and the interface state, etc., all need further research.

In conclusion, the performance of V-TFT can be improved by looking at the structure of the device, the preparation process, and the choice of active layer materials. V-TFT can be used in a wide range of applications, such as new displays, integrated circuits, sensors and flexible applications, due to its unique advantages. In the future, with the continuous maturity and improvement of vertical channel TFT technology, it is expected to become an important device replacing traditional planar TFT. It will be widely used in the commercialization of ultra-high-resolution and flexible electronic sensing.

## Figures and Tables

**Table 1:** Spacer material evaluation indicators for selection.

Spacer Material Selection Indicator	Material Types	Process Compatibility	Interface Properties	Cost Controllability
Selection requirements	Good insulating properties	Compatible with the other material preparation process	Minimize interface effects such as interface defects	The preparation process reproducible, large areas and low cost

**Table 2:** Data Comparison of Key Parameters of V-TFT Devices in Recent Years.

Years	Mobility (cm^2^/Vs)	I_on_/I_off_	SS (mV/dec)	Channel Length (nm)	Active Layer Materials	Method	Reference
2008	N/A	10^8^	800	100	a-Si:H	sputtering	[44]
2012	0.03	10^4^	3000–6000	310	IGZO	sputtering	[57]
2012	12.7	5 × 10^7^	70	500	ZnO	ALD	[51]
2013	3.96	10^3^	3600	1000	Poly silicon	sputtering	[46]
2013	11.8	2 × 10^7^	600	500	IGZO	sputtering	[58]
2014	3.3	8.8 × 10^6^	400	500	ZnO	ALD	[35]
2015	0.2	10^4^	400	300	IGZO	sputtering	[59]
2017	7.1	10^3^	1200	300	IGZO	ALD	[28]
2018	N/A	10^5^	900	121	In_2_O_3_	solution	[60]
2019	5.96	3.3 × 10^7^	210	300	ITO-stabilized ZnO	sputtering	[56]
2021	3.21	6.9 × 10^7^	460	250	IGZO	ALD	[36]
2021	0.1	8.8 × 10^3^	800	160	IGZO	ALD	[41]
2021	0.19	10^6^	540	130	IGZO	ALD	[27]
2022	2.2	4.2 × 10^9^	430	170	IGZO	ALD	[54]
2022	24.1	1.2 × 10^9^	213	400	IGZO	ALD	[29]
2023	N/A	4.8 × 10^9^	180	150	IGZO	ALD	[55]

**Table 4:** Charge transport properties of V-TFT compared with Planar TFT.

Device Type	Mobility (cm^2^/Vs)	I_on_/I_off_	SS (mV/dec)	Reference	Device Dimension	Power Consumption
Planar TFT	8.82	1.07 × 10^9^	330	[85]	Large	High
	11.5	2.4 × 10^10^	200	[86]		
	16.4	1.1 × 10^10^	340	[87]		
	10.8	9.2 × 10^7^	226	[88]		
	36.187	3.56 × 10^7^	315.45	[89]		
	12.7	7.6 × 10^5^	340	[90]		
	45.3	10^8^	210	[91]		
Vertical TFT	0.2	10^4^	400	[59]	Small	Low
	7.1	10^3^	1200	[26]		
	3.21	6.9 × 10^7^	460	[36]		
	0.1	8.8 × 10^3^	800	[41]		
	0.19	10^6^	540	[27]		
	2.2	4.2 × 10^9^	430	[54]		
	24.1	1.2 × 10^9^	213	[29]		

## Data Availability

Not applicable.
